# Prediction of local recurrence in tenosynovial giant cell tumor of the knee: Based on preoperative MRI evaluation into disease subtypes and severity

**DOI:** 10.1371/journal.pone.0287028

**Published:** 2023-06-14

**Authors:** Jun-Ho Kim, Seul Ki Lee, Jee-Young Kim

**Affiliations:** 1 Department of Orthopaedic Surgery, Center for Joint Diseases, Kyung Hee University Hospital at Gangdong, Seoul, Republic of Korea; 2 Department of Radiology, St. Vincent’s Hospital, College of Medicine, The Catholic University of Korea, Seoul, Republic of Korea; Villa Erbosa, Gruppo San Donato., ITALY

## Abstract

**Objective:**

Tenosynovial giant cell tumors (TSGCTs) of the knee differ in their clinical outcome according to disease subtypes and severity. The aim of this study was to determine the predictive MRI features related to local recurrence in TSGCT of the knee regarding disease subtypes and severity.

**Methods:**

This retrospective study included 20 patients with pathology-proven TSGCT of the knee who underwent preoperative MRI and surgery from Jan. 2007 to Jan. 2022. The anatomical point of the lesion was determined with a knee mapping. And then MRI features related to disease subtype including nodularity (single vs. multinodular); margin (circumscribed vs. infiltrative); peripheral hypointenseity (present vs. absent); internal hypointensity reflecting hemosiderin deposition (speckled vs. granular) were assessed. Third, MRI features related to disease severity including involvement of bone, cartilage, and tendon were evaluated. MRI features for predicting local recurrence of TSGCT were tested using chi-square test and logistic regression analysis.

**Results:**

Ten patients with diffuse-type TSGCT (D-TSGCT) and 10 patients with localized-type TSGCT (L-TSGCT) were included. There were six cases of local recurrence and all of them were D-TSGCT and none for L-TSGCT with statistical difference (*P* = 0.015). D-TSGCT that was direct risk factor for local recurrence showed more multinodular (80.0% vs. 10.0%; *P* = 0.007), infiltrative margin (90.0% vs. 10.0%; *P* = 0.002), and absent peripheral hypointensity (100.0% vs. 20.0%; *P* = 0.001) than L-TSGCT. Multivariate analysis showed infiltrative margin (odds ratio [OR], 81.0; *P* = 0.003) was independent MRI factor for D-TSGCT. Disease severity for risk of local recurrence included cartilage (66.7% vs. 7.1%; *P* = 0.024) and tendon (100.0% vs. 28.6%; *P* = 0.015) involvement compared to no local recurrence. Multivariate analysis showed tendon involvement (OR, 12.5; P = 0.042) was predictive MRI parameter for local recurrence. By combining tumor margin and tendon involvement, local recurrence was predicted sensitively on preoperative MRI (sensitivity, 100%; specificity, 50%; accuracy, 65%).

**Conclusion:**

D-TSGCTs was associated with local recurrence and showed multinodularity infiltrative margin, and absent peripheral hypointensity. Disease severity including cartilage and tendon involvement was associated with local recurrence. Preoperative MRI evaluation by combining disease subtypes and severity can predict local recurrence sensitively.

## Introduction

Tenosynovial giant cell tumors (TSGCTs), formerly known as pigmented villonodular synovitis, are locally aggressive neoplasms composed of synovial-like mononuclear cells mixed with multinuclear giant cells, foam cells, siderophages, and inflammatory cells that can involve the synovial tissue of tendon sheaths, bursae, and joints [1, 2]. They are classified as localized-type TSGCT (L-TSGCT) or diffuse-type TSGCT (D-TSGCT) according to their growth pattern based on the World Health Organization Classification (WHO) of Tumors of Soft Tissue and Bone, 2013 version are reiterated in the 2020 WHO classification [3, 4]. Although TSGCTs can occur in any part, they usually occur in the knee (>75% of cases) [5, 6]. TSGCTs differ in their clinical presentation, treatment options, response to treatment, and prognosis according to disease subtypes [3]. D-TSGCT is known to have a high rate of recurrence (8%-56%) after surgery [7–10]. Extensive surgical resection of synovial tumorous tissues has long been the standard therapy for D-TSGCT around the knee, with procedures including arthroscopic or open synovectomy, and adjuvant therapies such as radiation therapy and immunotherapy are recommended [11–14]. In the case of extraarticular D-TSGCT of the knee, it is more like to be resected incompletely, leading to high recurrence rates, and adjuvant treatment or further surgery is often needed to treat residual disease [13, 15, 16].

Previously published studies have mainly focused on disease extent or severity of TSGCT around the knee and these findings may provide important information for deciding surgical extent and be related to clinical outcome [16–22]. However, little effort has been made to determine MRI features that can differentiate disease subtypes around the knee which is directly associated with a risk of local recurrence [23–25]. If the lesion is D-TSGCT, it is likely to have severer extent compared to L-TSGCT, but not always and moreover, it is not easy to classify disease subtypes of TSGCT only by morphology on preoperative MRI [26].

Since both disease subtypes and disease severity are important for clinical outcome, we designed this study to determine the predictive MRI features related to local recurrence in TSGCT of the knee regarding disease subtypes and severity.

## Materials and methods

### Study population

This retrospective study was approved by St. Vincent’s Hospital’s Institutional Review Board, and the requirement for informed consent was waived. TSGCT cases of the knee were enrolled from January 2007 to January 2022 according to the following inclusion criteria: patients underwent (1) preoperative MRI of the knee and (2) surgery. Only newly diagnosed, histologically confirmed TSGCTs of the knee were included. In total, 20 patients with TSGCTs of the knee were finally enrolled. Demographic findings, including sex, age, and follow-up data more than 1 year were obtained from the medical records.

### MRI acquisition

MRI was performed with 1.5-T (Ingenia, Philips Healthcare, The Netherlands) 3.0-T (Magnetom Verio, Siemens Healthineers, or Ingenia, Philips Healthcare) scanners. MRI protocols included spin-echo T1-weighted (TR/TE range, 370–693/10-19 in 1.5-T, 623/11 in 3.0-tesla), spin-echo T2-weighted with and without fat suppression (TR range/TE range: 1648–3280/80–100 in 1.5-tesla, 4000–6200/63–76 in 3.0-tesla), and gadolinium enhancement of T1-weighted images with fat suppression. The imaging protocol varied between examinations, with or without the addition or a gradient-recalled echo pulse sequence by the supervising radiologists. Axial, coronal, and sagittal images were obtained for all patients. Gadolinium-enhanced T1-weighted images were obtained in at least two orthogonal planes.

### MRI analysis

**Fig 1:**
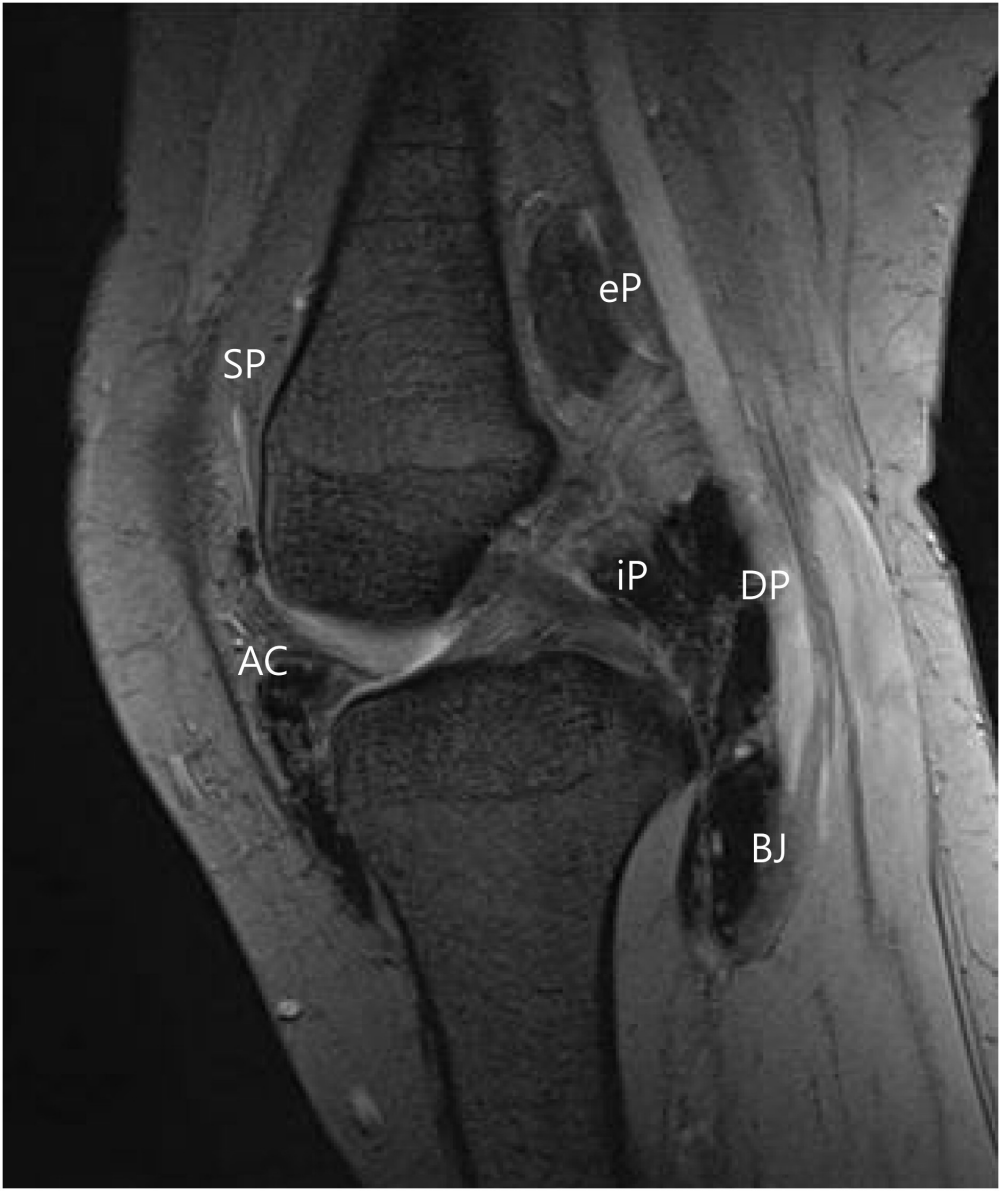
The mapping scheme for localization of TSGCT. The intraarticular space is divided into three points including SP (suprapatellar pouch), AC (anterior compartment), and iP (posterior compartment). The extraarticular space is divided into three points including eP (posterior to joint capsule), DP (direct posterior to joint capsule), and BJ (below the joint capsule).

**Fig 2:**
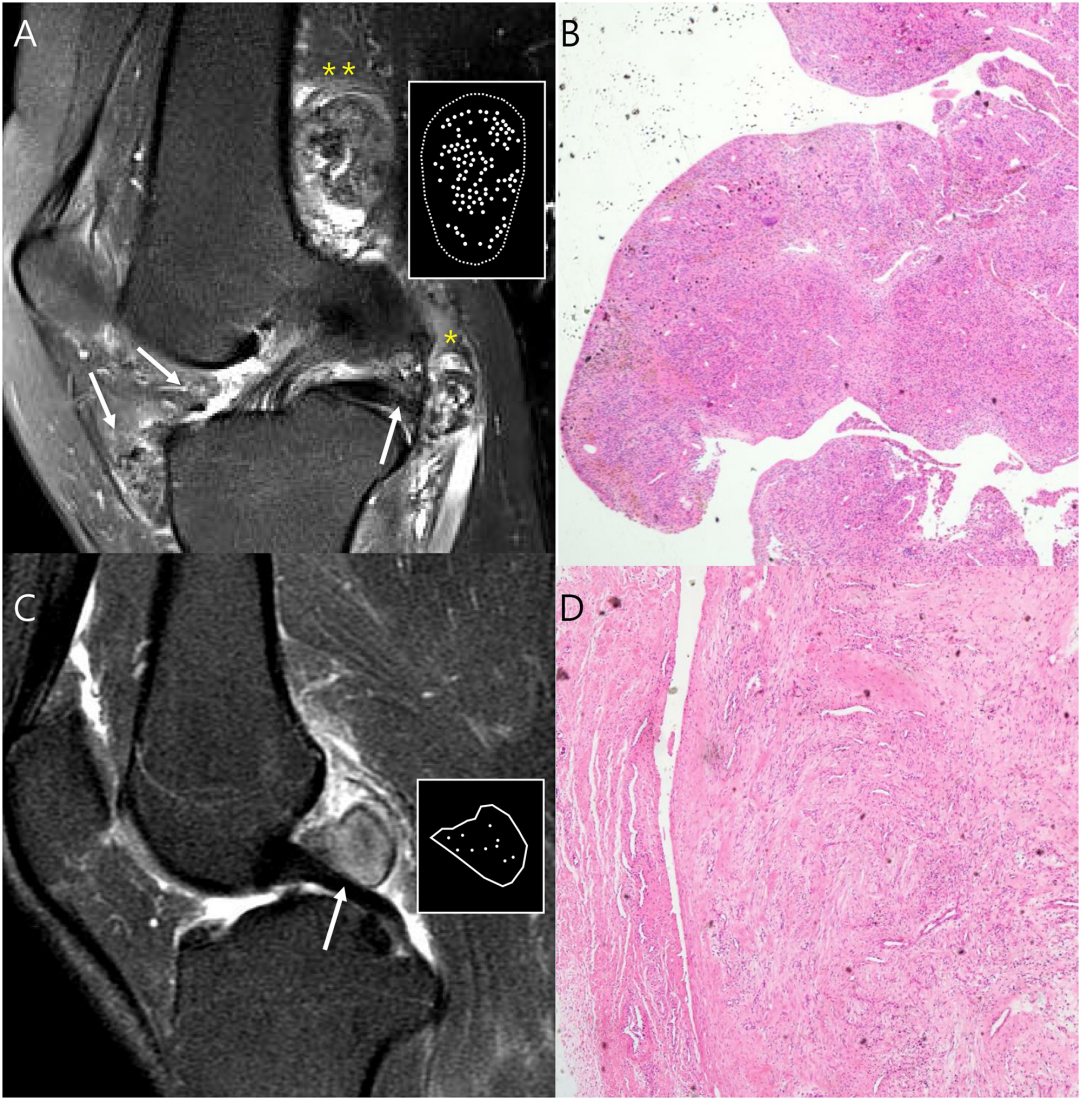
Definition of the MRI features relating to the disease subtypes. (A) Pathology-proven D-TSGCT shows that the intraarticular mass (iP, arrow) extending to subpopliteal recess (DP, *), and some parts have crossed to an extraarticular location (eP, **). The masses contain two or more distinct nodules, classified into type II nodularity with infiltrative margin from surrounding tissues. The representative mass (**) shows the granular internal hypointensity without peripheral hypointensity. (B) This mass shows an villonodular and infiltrative margin and abundant hemosiderin-laden macrophages through the tumor (H&E, X40). (C) Pathology-proven L-TSGCT shows the intraarticular (iP, arrow) single nodule, classified into type I nodularity. The mass shows a circumscribed margin from surrounding tissues and a speckled internal hypointensity with peripheral hypointensity. (D) This mass shows a well-circumscribed mass entirely enveloped by a thin fibrous septa and a scant amount of hemosiderin deposit (H&E, X40).

All images were independently reviewed by 2 musculoskeletal radiologists with 7 and 26 years of clinical experience of musculoskeletal radiology, respectively; both radiologists were blinded to the pathologic and intraoperative findings. First, the number of anatomical point of the lesion was determined with a knee mapping on MRI scans. The anatomical point of the lesion was analyzed by modifying the mapping scheme devised by Kim et al. [16] (Fig 1). Second, MRI features relating to the disease subtypes, including the nodularity), margin, peripheral hypointensity, and morphology of the internal hypointensity, were assessed [26] (Fig 2). The nodularity was established according to the Al-Qattan classification as either type I (single), i.e., a single round or multilobulated tumor, or type II (multinodular), i.e., exhibiting ≥2 distinct, separated tumors [27, 28]. The margin was classified as circumscribed when the border was clearly delineated from surrounding structures or infiltrative if the borders were indistinguishable with surrounding structures [29]. The peripheral hypointensity was evaluated on T2-weighted image and defined as presence or absence. The morphology of internal hypointensity was also evaluated on T2-weighted image and defined as having a speckled (defined as loosely scattered, low-signal foci) or granular (defined as densely clustered, low-signal dots) appearance, representing the density of hemosiderin deposition [26, 30]. Third, MRI features related to disease severity including involvement of bone, cartilage, and tendon were also evaluated.

### Statistical analysis

Student’s *t*-test was performed to compare ages, and other continuous values, while the chi-squared test was used to compare the two groups in terms of sex and other categorical values. MRI features for predicting local recurrence of TSGCT were tested using multivariate logistic regression analysis. Interobserver agreement and agreement between different modalities were assessed using kappa statistics. Data were analyzed using MedCalc statistical software version 11.5.1.0 (MedCalc Software bvba, Ostend, Belgium). *P*-value < 0.05 was considered to be statistically significant.

## Results

### Patient characteristics

Patients were divided into 10 D-TSGCT (mean age, 33.5 ± 12.0 years; age range, 26–62 years; 7 women) and 10 L-TSGCT (mean age, 33.4 ± 13.7 years; age range, 19–59 years; 4 women). There were no differences in age (*P* = 0.966) and gender (*P* = 0.369) between disease subtypes. There was no difference in number of lesion between L-TSGCT (1 point, n = 9; 2 points, n = 0; 3 points, n = 1; 4 points, n = 0) and D-TSGCT (1 point, n = 5; 2 points, n = 4; 3 points, n = 0; 4 points, n = 1, *P* = 0.067).

### MRI features regarding disease subtypes

**Fig 3:**
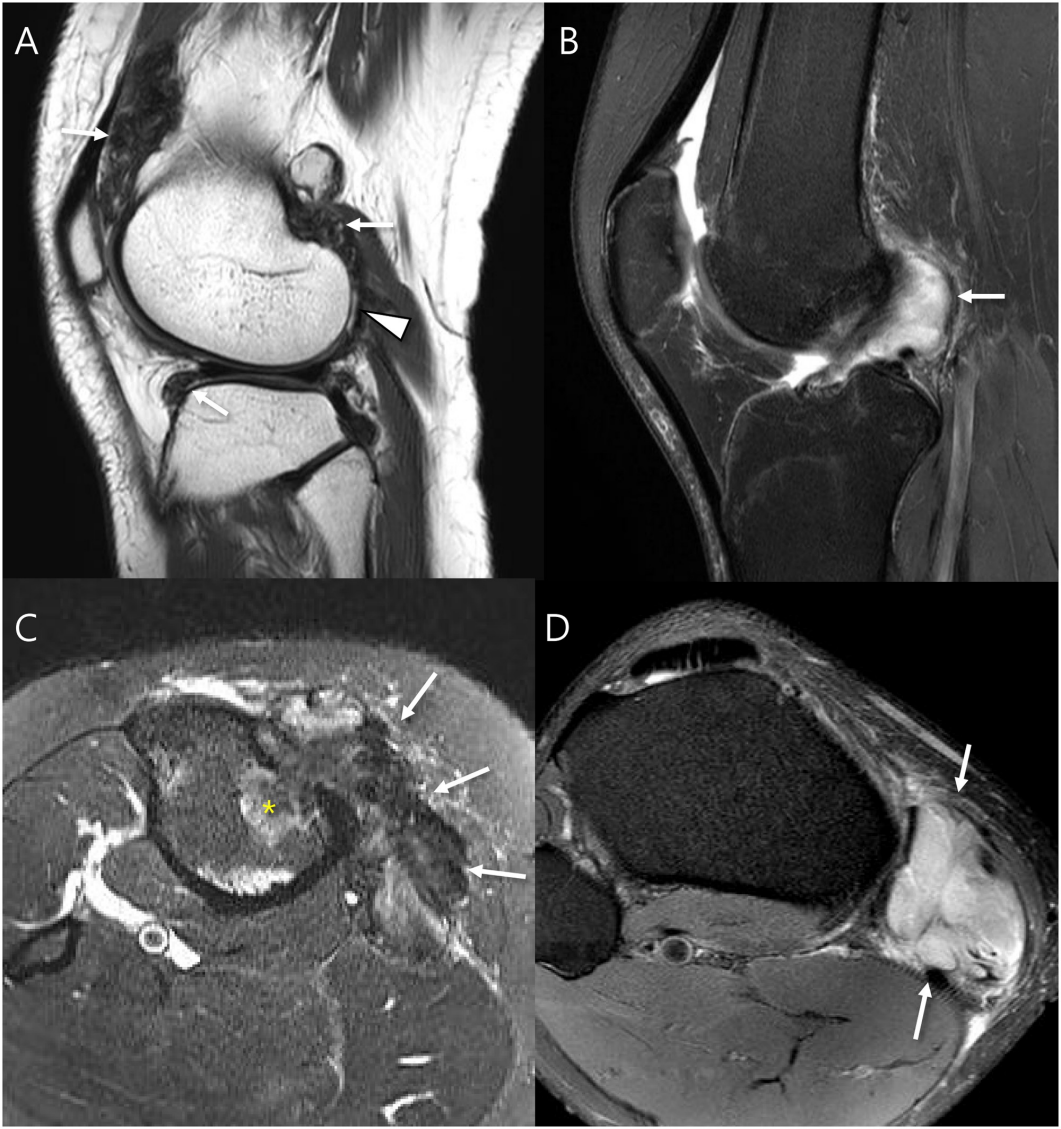
MRI features regarding disease subtypes. (A) Sagittal T2-weighted image of a 24-year old woman with intraarticular D-TSGCT shows soft tissue masses that are very low signal intensity located in knee joint (arrows) showing infiltrative margin and containing granular internal hypointensity without visible peripheral hypointensity. Note that the lateral femoral condyle cartilage injury is combined (arrowhead). (B) Sagittal T2-weighted image with fat suppression of a 26-year old woman with intraarticular L-TSGCT shows soft tissue mass at the posterior femoral recess (arrow). Note that the mass shows a circumscribed margin and contains a speckled internal hypointensity with peripheral hypointensity. (C) Axial T2-weighted image with fat suppression of a 40-year old woman with extraarticular D-TSGCT shows soft tissue masses that are very low signal intensity at the insertion of the pes anserinus conjoined tendon (arrows) presenting the multinodular masses with infiltrative margin with bone invasion (*) and containing granular internal hypointensity (arrows) without visible peripheral hypointensity. (D) Axial T2-weighted image with fat suppression of a 30-year old man with extraarticular L-TSGCT shows soft tissue masses along the pes anserinus conjoined tendon (arrows) presenting a circumscribed margin and containing a speckled internal hypointensity (arrows) with peripheral hypointensity.

There were six cases of local recurrence (median time, 2 years; range, 0.5–10 years) and all of them were D-TSGCT and none for L-TSGCT with statistical difference (*P* = 0.015). The corresponding MRI features of multinodularity (80.0% vs. 10.0%; *P* = 0.007), infiltrative margin (90.0% vs. 10.0%; *P* = 0.002), and absent peripheral hypointensity (100.0% vs. 20.0%; *P* = 0.001) were significantly more common in D-TSGCT cases compared to L-TSGCT cases (Fig 3 and Table 1).

**Table 1:** MRI findings between L-TSGCT and D-TSGCT.

	L-TSGCT	D-TSGCT	*P* value
	(n = 10)	(n = 10)	
Nodularity			0.007
• single	9 (90.0%)	2 (20.0%)	
• multinodular	1 (10.0%)	8 (80.0%)	
Margin			0.002
• circumscribed	9 (90.0%)	1 (10.0%)	
• infiltrative	1 (10.0%)	9 (90.0%)	
Peripheral hypointensity			0.001
• presence	2 (20.0%)	10 (100.0%)	
• absence	8 (80.0%)	0 (0.0%)	
Internal hypointensity			0.180
• speckled	7 (70.0%)	3 (30.0%)	
• granular	3 (30.0%)	7 (70.0%)	

**Fig 4:**
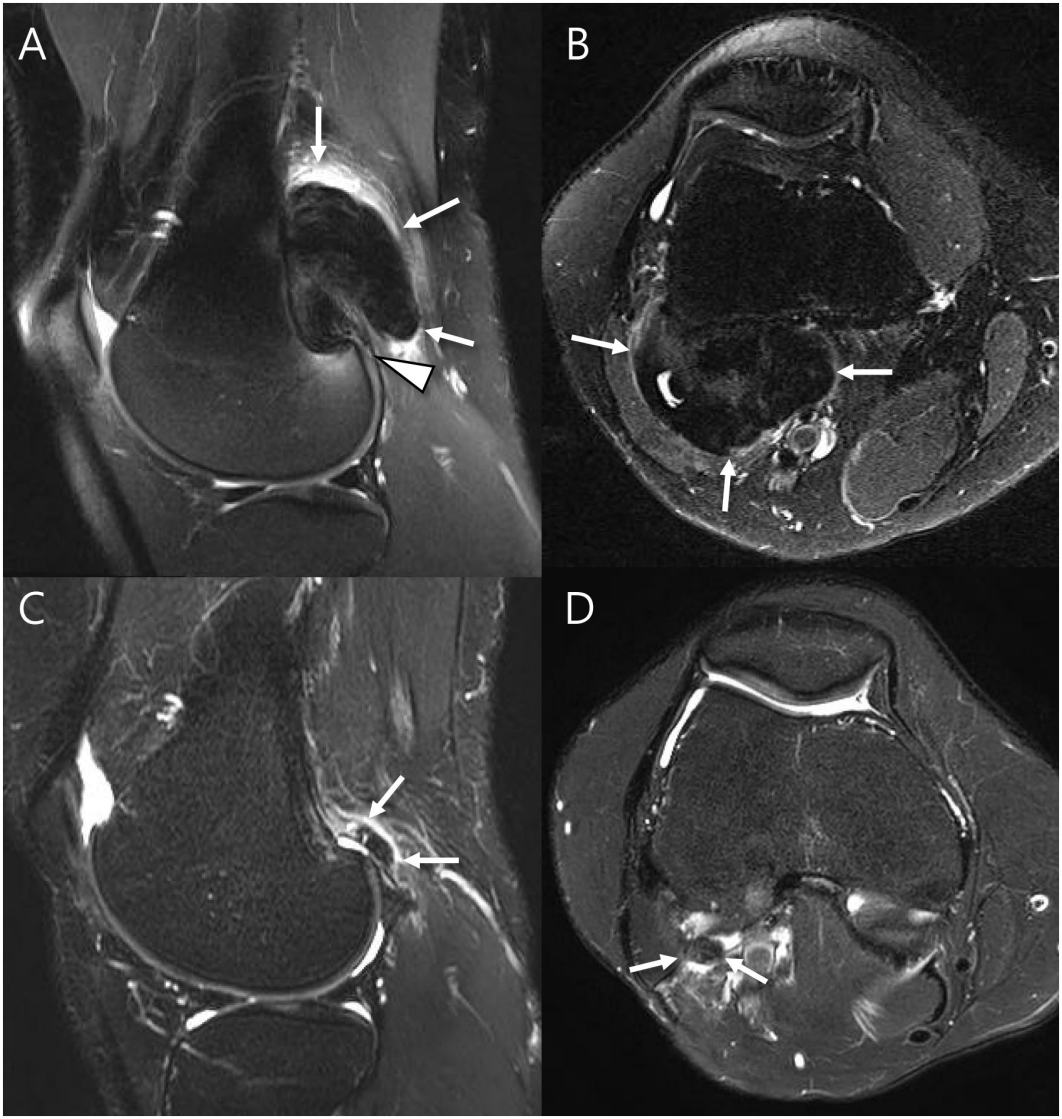
A 29-year old woman with pathology-proven extraarticular D-TSGCT of knee. (A and B) Preoperative sagittal and axial T2-weighted images with fat suppression show soft tissue mass that are very low signal intensity located in the extraarticular posterolateral aspect of knee with infiltrative margin containing granular internal hypointensity without visible peripheral hypointensity (arrows). Note that the lateral femoral condyle cartilage is involved by tumor (arrowhead). (C and D) Follow-up MRI after 30 months show recurrent mass with same nature (arrows).

Multivariate analysis showed infiltrative margin (odds ratio [OR], 81.0; *P* = 0.003) was independent MRI factor for D-TSGCT (Fig 4 and Table 2).

**Table 2:** Logistic regression analysis of MRI parameters predicting D-TSGCT.

	Univariate				Multivariate			
	β	Odds ratio	95% CI	*P*-value	β	Odds ratio	95% CI	*P*-value
Nodularity	3.584	36.000	2.7–476.2	0.007	-	-	-	-
Margin	4.394	81.0000	4.3–1504.4	0.003	4.394	81.0000	4.3–504.4	0.003
Peripheral hypointensity	-3.584	0.028	0.002–0.367	0.007	-	-	-	-
Internal hypointensity	1.695	5.444	0.8–36.8	0.021	-	-	-	-

### MRI features regarding disease severity

Disease severity including cartilage (66.7% vs. 7.1%; *P* = 0.024) and tendon (100.0% vs. 28.6%; *P* = 0.015) involvement were frequently noted in recurrence cases compared to no recurrence cases (Fig 4 and Table 3).

**Table 3:** MRI findings regarding disease severity associated with local recurrence.

	No recurrence	Local recurrence	*P* value
	(n = 14)	(n = 6)	
Bone			0.682
• no involvement	10 (71.4%)	3 (50.0%)	
• involvement	4 (28.6%)	3 (50.0%)	
Cartilage			0.024
• no involvement	13 (92.9%)	2 (33.3%)	
• involvement	1 (7.1%)	4 (66.7%)	
Tendon			0.015
• no involvement	10 (71.4%)	0 (0.0%)	
• involvement	4 (28.6%)	6 (100.0%)	

**Fig 5:**
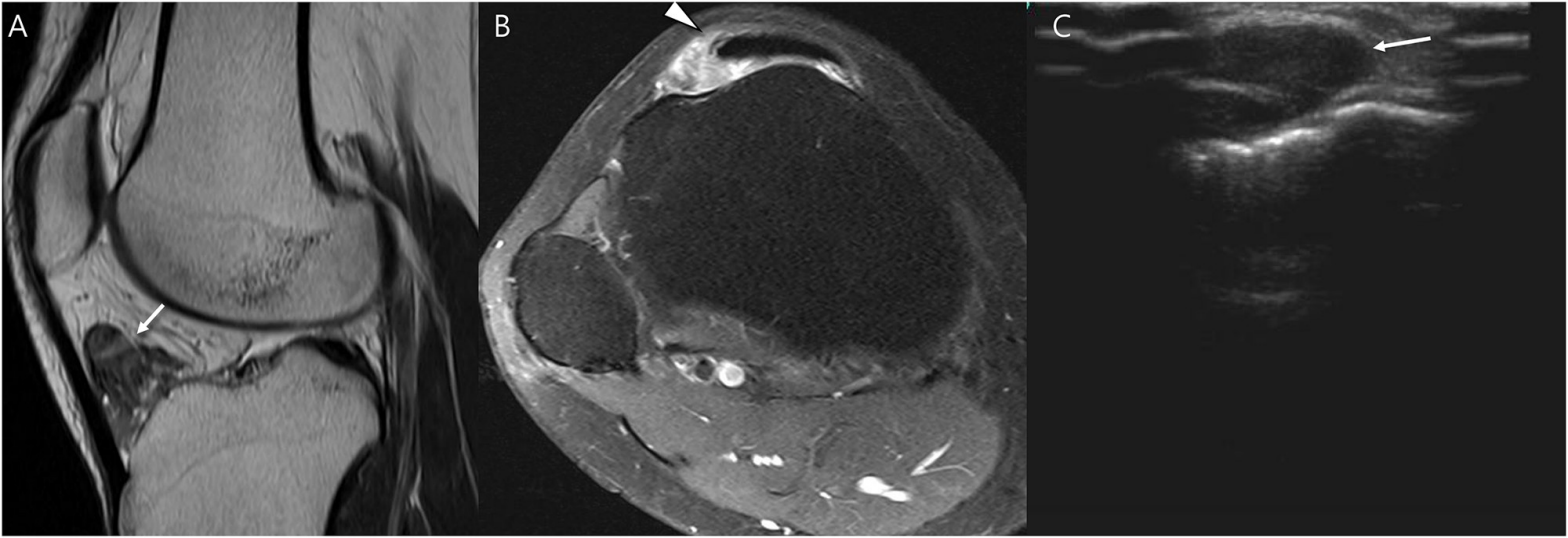
A 19-year old woman with pathology-proven intraarticular L-TSGCT of knee. (A and B) Preoperative sagittal and axial T2-weighted images show soft tissue mass located in the Hoffa’s fat pad with circumscribed margin containing granular internal hypointensity without visible peripheral hypointensity (arrow). Note that the patellar tendon is involved by tumor (arrowhead). (C) Follow-up ultrasound after 13 months show recurrent mass (arrow).

**Fig 6:**
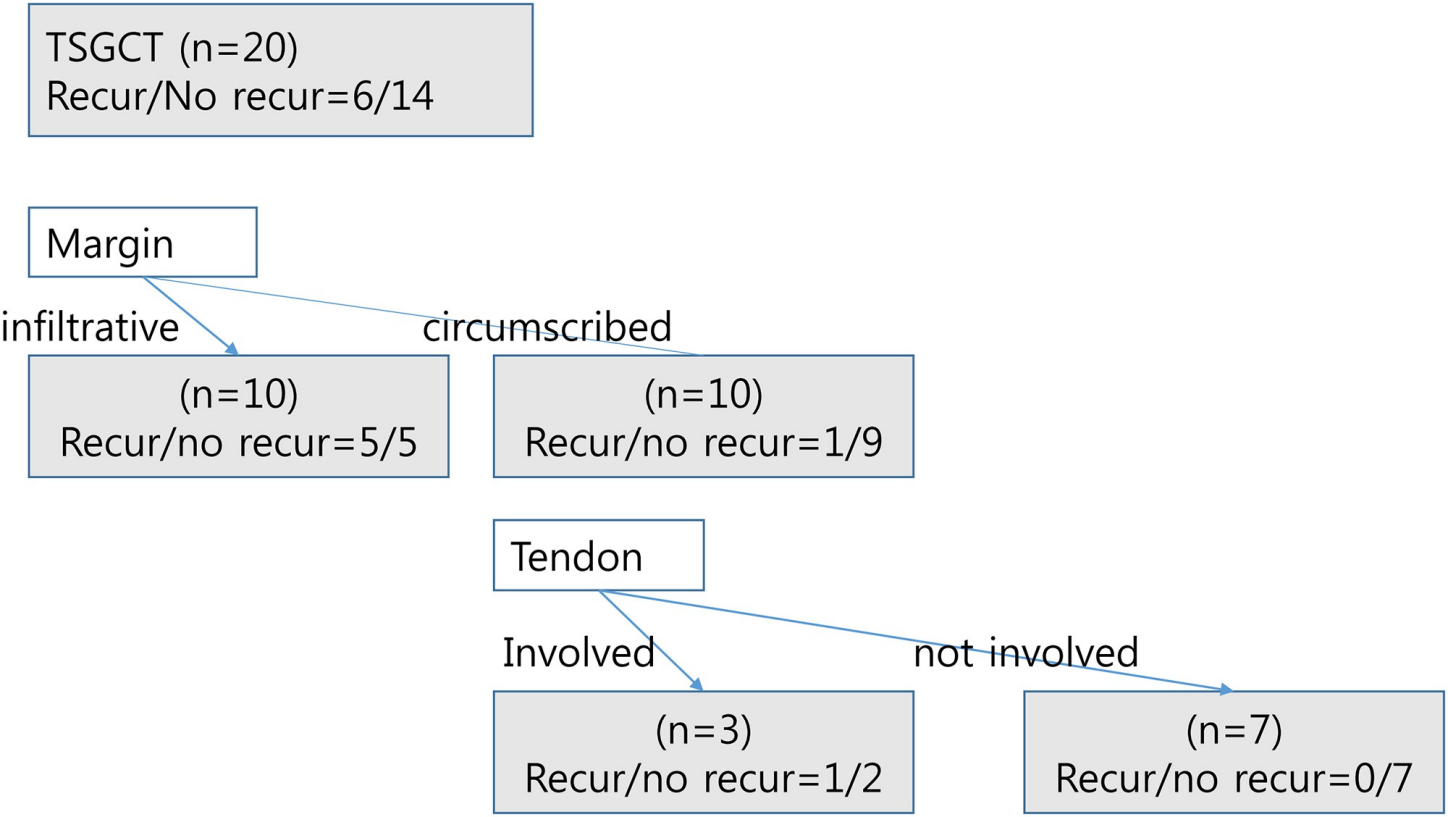
MRI parameters tree to predict local recurrence. MRI parameters begin with the margin followed by tendon involvement with sensitivity, 100%; specificity, 50%; accuracy, 65%.

Multivariate analysis showed tendon involvement (OR, 12.5; *P* = 0.042) was predictive MRI parameter for local recurrence (Fig 5 and Table 4). By combining tumor margin and tendon involvement, local recurrence was predicted sensitively on preoperative MRI (Fig 6, sensitivity, 100%; specificity, 50%; accuracy, 65%).

**Table 4:** Logistic regression analysis of MRI parameters predicting local recurrence.

	Univariate				Multivariate			
	β	Odds ratio	95% CI	*P*-value	β	Odds ratio	95% CI	*P*-value
Bone	0.916	2.500	0.3–18.0	0.363	-	-	-	-
Cartilage	0.916	2.5000	0.3–18.0	0.363	-	-	-	-
Tendon	2.526	12.500	1.0–143.4	0.042	2.526	12.500	1.0–143.4	0.042

Interobserver agreement ranged between κ of 0.530 and 0.875 for MRI features.

## Discussion

We found that D-TSGCTs was associated with local recurrence and showed multinodularity infiltrative margin, and absent peripheral hypointensity. Disease severity including cartilage and tendon involvement was associated with local recurrence. Preoperative MRI evaluation by combining infiltrative margin and tendon involvement can predict local recurrence sensitively.

Preoperative classification of TSGCT of the knee is important in decision-making. First of all, since TSGCTs are benign tumors with locally aggressive features, surgery should be planned while maintaining the balance between complete resection and knee function [1]. Because surgical resection of tumors is complicated and difficult, addition of multimodality approaches, such as external beam radiotherapy, radiation synovectomy, or colony-stimulating factor-1 receptor inhibitors, i.e., imatinib are is considered in unresectable cases [19, 31, 32]. When considering the use of radiotherapy as a treatment for TSGCT of the knee, it is important to take into account potential risks such as malignant transformation or post-operative complications (infection) [33]. Recent studies have shown that medical treatments can be effective in managing unresectable TSGCT, and in some cases, these treatments have demonstrated positive outcomes [34]. Therefore, preoperative classification plays a decisive role in determining the treatment options and predicting clinical outcome [1]. However, recent literature lacks MRI discriminating features to classify by disease subtype, and only a few papers have suggested an extent or severity classification of TSGCTs, with even fewer focusing on knee TSGCTs [1, 35]. Therefore, we focused on knee TSGCTs and classified them by subtype and severity to predict local recurrence.

Some studies have evaluated MRI features between D-TSGCT and L-TSGCT among intraarticular TSGCT of knee [10, 36]. Nguyen et al. [10] compared MRI signal intensities, joint effusion, osseous changes, chondromalacia, juxtacapsular disease and concomitant joint involvement between two subtypes of the intraarticular TSGCT of knee, and reported that there was no significant difference in MRI signal intensities or other imaging findings except joint effusion [10]. Contrary to this study, our study analyzed various MRI features and presented that D-TSGCTs showed more multinodular, infiltrative margin with absent peripheral hypointensity, and associated with local recurrence. It seems that D-TSGCTs appeared as locally aggressive lesions characterized by multinodularity with infiltrative margin and no peripheral fibrous. The severity of TSGCTs may be evaluated by casting molds or invasion into nearby structures [30]. The cartilage and tendon involvement showed statistical significance (*P*<0.05) between local recurrence and no recurrence cases. More severe disease extent of cartilage and tendon involvement showed association with local recurrence, even if in L-TSGCTs [26]. Although adjacent structures involvement such as bone invasion might be overlapping feature in both D-TSGCT and L-TSGCT, combining analysis of MRI features regarding disease subtypes and severity could be helpful in determining the surgical plan to predict local recurrence.

The limitations of this study include its use of a relatively small sample size from a single institution and a retrospective design. Second, we did not include gadolinium enhanced sequences in the image analysis because TSGCT revealed variable degrees of enhancement in previous studies [37, 38] and no statistical differences and our pilot study.

## Conclusions

Preoperative MRI depicts distinguishable features between L-TSGCT and D-TSGCT of the knee and D-TSGCT is associated with local recurrence. Discriminating features D-TSGCTs appear to be multinodular masses with infiltrative margin and absent peripheral hypointensity. More severe disease extent of cartilage and tendon involvement showed association with local recurrence. Combining MRI analysis regarding disease subtypes and severity could be helpful in determining the surgical plan to predict local recurrence during follow-up.

## Supporting information

S1 File This is the data sheet. (CSV)
